# Epidemiology of HBoV1 infection and relationship with meteorological conditions in hospitalized pediatric patients with acute respiratory illness: a 7-year study in a subtropical region

**DOI:** 10.1186/s12879-018-3225-3

**Published:** 2018-07-16

**Authors:** Wen-Kuan Liu, Qian Liu, De-Hui Chen, Wei-Ping Tan, Yong Cai, Shu-Yan Qiu, Duo Xu, Chi Li, Xiao Li, Zheng-Shi Lin, Rong Zhou

**Affiliations:** 1 State Key Laboratory of Respiratory Diseases, National Clinical Research Center for Respiratory Disease, The First Affiliated Hospital of Guangzhou Medical University, Guangzhou Institute of Respiratory Health, Guangzhou Medical University, Guangzhou, China; 2 Central Laboratory, The First Affiliated Hospital of Guangdong Pharmaceutical University, Guangzhou, China; 3 Sun Yat-Sen Memorial Hospital, Sun Yat-Sen University, Guangzhou, China

**Keywords:** Human bocavirus 1, Acute respiratory illness, Epidemiology, Meteorological conditions

## Abstract

**Background:**

Human bocavirus 1 (HBoV1) is an important cause of acute respiratory illness (ARI), yet the epidemiology and effect of meteorological conditions on infection is not fully understood. To investigate the distribution of HBoV1 and determine the effect of meteorological conditions, hospitalized pediatric patients were studied in a subtropical region of China.

**Methods:**

Samples from 11,399 hospitalized pediatric patients (≤14 years old), with ARI were tested for HBoV1 and other common respiratory pathogens using real-time PCR, between July 2009 and June 2016. In addition, local meteorological data were collected.

**Results:**

Of the 11,399 patients tested, 5606 (49.2%) were positive for at least one respiratory pathogen. Two hundred forty-eight of 11,399 (2.2%) were positive for HBoV1 infection. Co-infection was common in HBoV1-positive patients (45.2%, 112/248). A significant difference in the prevalence of HBoV1 was found in patients in different age groups (*p* < 0.001), and the peak prevalence was found in patients aged 7–12 months (4.7%, 56/1203). Two HBoV1 prevalence peaks were found in summer (between June and September) and winter (between November and December). The prevalence of HBoV1 was significantly positively correlated with mean temperature and negatively correlated with mean relative humidity, and the mean temperature in the preceding month had better explanatory power than the current monthly temperature.

**Conclusions:**

This study provides a better understanding of the characteristics of HBoV1 infection in children in subtropical regions. Data from this study provide useful information for the future control and prevention of HBoV1 infections.

## Background

Human bocavirus 1 (HBoV1), which belongs to family *Parvoviridae*, was firstly identified in respiratory secretions of children with respiratory tract disease in 2005 [1, 2]. HBoV1 has been confirmed as an important respiratory pathogen and is found in respiratory infections in children and adults worldwide. The prevalence of HBoV1 nucleic acid detection varies from 1.5 to 33% in patients with acute respiratory illness (ARI), according to different studies [3–7]. Serological and nucleic acid test results are generally consistent [8–11], showing HBoV1 infection is very common. HBoV1 can cause both upper respiratory illness (URI) and lower respiratory illness (LRI) [12–18]. Infection with HBoV1 can lead to development of a cough, rhinitis, fever and other common clinical symptoms [15, 19]. In some cases, it can cause respiratory distress, hypoxia, wheezing and other severe respiratory symptoms [18, 20]. Clinical diagnosis is mainly pneumonia, bronchitis, pneumothorax, mediastinal emphysema and otitis media and other complications [18–22]. In some cases, patients develop severe respiratory injury symptoms, which can be fatal [21, 23]. HBoV1 can be detected in fecal samples [24], blood samples [25, 26], urine [27, 28], cerebrospinal fluid [29–31], river water [32] and sewage [33, 34], indicating that HBoV1 may be associate with a variety of diseases. Current in vitro studies modeling tissue-like airway epithelial cells cultures show HBoV1 infection can lead to disruption of the tight-junction barrier, loss of cilia and epithelial cell hypertrophy [35–37], similar to lung injury tissue changes in vivo. There is currently no vaccine or specific treatment for this virus; prevention and treatment of HBoV1-related diseases still require further research. The prevalence of respiratory viruses is associated with many factors, including local climate, which may impact the survival and spread of the viruses [38]. Studying the epidemiology of HBoV1 and its relationship with meteorological conditions will improve diagnosis, treatment, control and prevention of this virus.

In this study, we investigated the epidemiology of HBoV1 infection in children (≤14 years old) hospitalized with ARI in a subtropical region in China over a 7-year period. In addition, we collected climate data to determine if there was a relationship between HBoV1 prevalence and meteorological conditions. This study will add to existing epidemiological data on HBoV1 and its relationship with climate conditions in subtropical regions and will play a positive role in HBoV1 control and prevention.

## Methods

### Respiratory sample and meteorological data collection

The study sites were three tertiary hospitals in Guangzhou, southern China (Longitude: E112° 57′ to E114 03′; Latitude N22° 26′ to N23° 56′). Inclusion criteria were pediatric patients (≤14 years old) who presented with at least two of the following symptoms: cough, pharyngeal discomfort, nasal obstruction, rhinitis, dyspnea or who were diagnosed with pneumonia by chest radiography during the previous week. Chest radiography was conducted according to the clinical situation of the patient. Throat swab samples were collected from the enrolled patients between July 2009 and June 2016 for routine screening for respiratory viruses, *Mycoplasma pneumoniae* (MP), and *Chlamydophila pneumoniae* (CP). The samples were refrigerated at 2–8 °C in viral transport medium, transported on ice and analyzed immediately or stored at − 80 °C before analysis, as described previously [15, 39].

Meteorological data for Guangzhou, were collected from July 2009 to June 2016, from the China Meteorological Administration, including the monthly mean temperature (°C), mean relative humidity (%), rainfall (mm), mean wind speed (m/s), mean air pressure (hPa), mean vapor pressure (hPa), sunshine duration (h).

### Real-time PCR for HBoV1 and common respiratory pathogen detection

DNA and RNA were extracted from the respiratory samples using the QIAamp DNA Mini Kit and QIAamp Viral RNA Mini Kit (Qiagen, Shanghai, China), respectively, in accordance with the manufacturer’s protocols. Taqman real-time PCR for HBoV1 was designed based on the conserved region of the NP1 gene, as described previously [15]. Common respiratory pathogens, including respiratory syncytial virus (RSV), influenza A virus (InfA), influenza B virus (InfB), four types of parainfluenza (PIV1–4), adenovirus (ADV), enterovirus (EV), human metapneumovirus (HMPV), four strains of human coronavirus (HCoV-229E, OC43, NL63 and HKU1), human rhinovirus (HRV), MP and CP were detected simultaneously as previously reported [40].

### Statistical analysis

Data were analyzed using Chi-squared test and Fisher’s exact test in SPSS 19.0 (SPSS Inc., Chicago, IL, USA). Correlation with climate data was analyzed using multiple linear regression analysis. All tests were two-tailed and a *p* value < 0.05 was considered as statistically significant.

## Results

### Patients and HBoV1 distribution

Eleven thousand three hundred ninety-nine pediatric patients (≤14 years old) hospitalized with ARI were enrolled in the study between July 2009 and June 2016. The male-to-female ratio was 1.82:1 (7361:4038) and the median age was 1.75 years (interquartile range 0.75–3.83). Overall, 86.5% (9857/11399) of patients were under the age of 5 years. All the 11,399 patients were tested for all 18 pathogens mentioned, and 5606 (49.2%) were positive for one or more of those pathogens (Table 1), and had a median age of 1.50 years (interquartile range 0.67–3.00). The male-to-female ratioes were 1.94: 1 (3698:1908) in pathogen-positive patients and 1.72: 1 (3663:2130) in pathogen-negative patients (*p* = 0.002).

**Table 1:** Distribution of respiratory pathogens in 11,399 pediatric patients hospitalized with acute respiratory illness

Pathogen	HBoV1	infA	infB	RSV	EV	HRV^a^	ADV	PIV1	PIV2	PIV3	PIV4	229E	OC43	NL63	HKU1	HMPV	MP	CP	Positive rate, %
HBoV1	248	13	4	29	15	17	14	4	3	10	1	0	9	3	1	7	14	0	2.2
infA		839	34	95	41	8	23	9	7	11	0	7	38	4	4	12	46	1	7.4
infB			300	25	9	6	4	1	1	6	0	0	9	0	1	5	15	2	2.6
RSV				1690	73	45	38	9	13	14	3	10	29	10	3	16	38	8	14.8
EV					498	16	24	14	2	13	1	4	10	6	1	10	21	5	4.4
HRV^a^						402	14	3	2	12	2	2	11	1	1	3	21	6	5.0
ADV							621	5	3	7	2	3	14	5	2	9	36	3	5.4
PIV1								116	2	2	0	2	5	1	0	1	9	0	1.0
PIV2									72	3	0	2	9	0	0	2	3	0	0.6
PIV3										296	0	1	15	1	1	3	15	1	2.6
PIV4											25	1	1	0	0	0	3	0	0.2
229E												64	14	2	0	3	3	0	0.6
OC43													346	2	1	14	27	3	3.0
NL63														60	1	1	3	1	0.5
HKU1															38	1	3	1	0.3
HMP																321	9	0	2.8
MP																	760	2	6.7
CP																		77	0.7
Single infection	136	546	203	1314	286	261	458	67	35	203	13	26	185	28	19	243	531	51	40.4
Co-infection	112	293	97	376	212	141	163	49	37	93	12	38	161	32	19	78	229	26	8.8

Data are number in each group, except where specifically stated. *HBoV1* human bocavirus 1, *InfA* influenza A virus, *InfB* influenza B virus, *RSV* respiratory syncytial virus, *EV* enterovirus, *HRV* human rhinovirus, *PIV1–4* parainfluenza 1–4, *ADV* adenovirus, *HMPV* human metapneumovirus, *229E* human coronavirus 229E, *OC43* human coronavirus OC43, *NL63* human coronavirus NL63, *HKU1* human coronavirus HKU1, *MP Mycoplasma pneumoniae*, *CP Chlamydophila pneumoniae*. ^a^HRV was detected since January 2012, and a total of 8084 cases were collected

Two hundred forty-eight of 11,399 patients (2.2%) tested positive for HBoV1 infection. Of the HBoV1-positive patients, 112 (45.2%) were co-infected with other pathogens, most frequently with RSV (11.7%, 29/248) (Table 1). The median age was 1 year (interquartile range 0.75–1.83). The male-to-female ratio was 2.54:1 (178:70) in HBoV1-positive patients and 1.81:1 (7183:3968) in HBoV1-negative patients (*p* = 0.019).

### Age distribution of HBoV1-positive patients

**Fig. 1:**
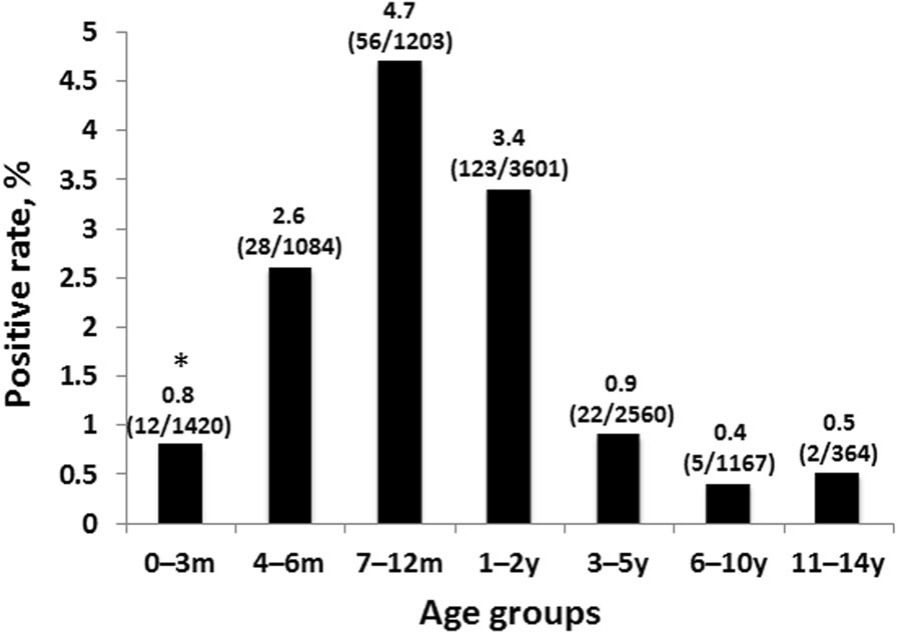
Age distribution of HBoV1 infection in 11,399 pediatric patients hospitalized with acute respiratory illness. *Date was presented as HBoV1 positive rate (no. of HBoV1-positive patients/no. of patients in each group); m: months; y: year(s)

To clarify the age distribution of HBoV1, patients were divided into seven age groups; 0–3 months, 4–6 months, 7–12 months, 1–2 years, 3–5 years, 6–10 years and 11–14 years old. There was a significant difference in the prevalence of HBoV1 in patients in different age groups (*p* < 0.001) and the peak prevalence was found in patients aged 7–12 months (4.7%, 56/1203) (Fig. 1).

### Seasonal distribution of HBoV1

**Fig. 2:**
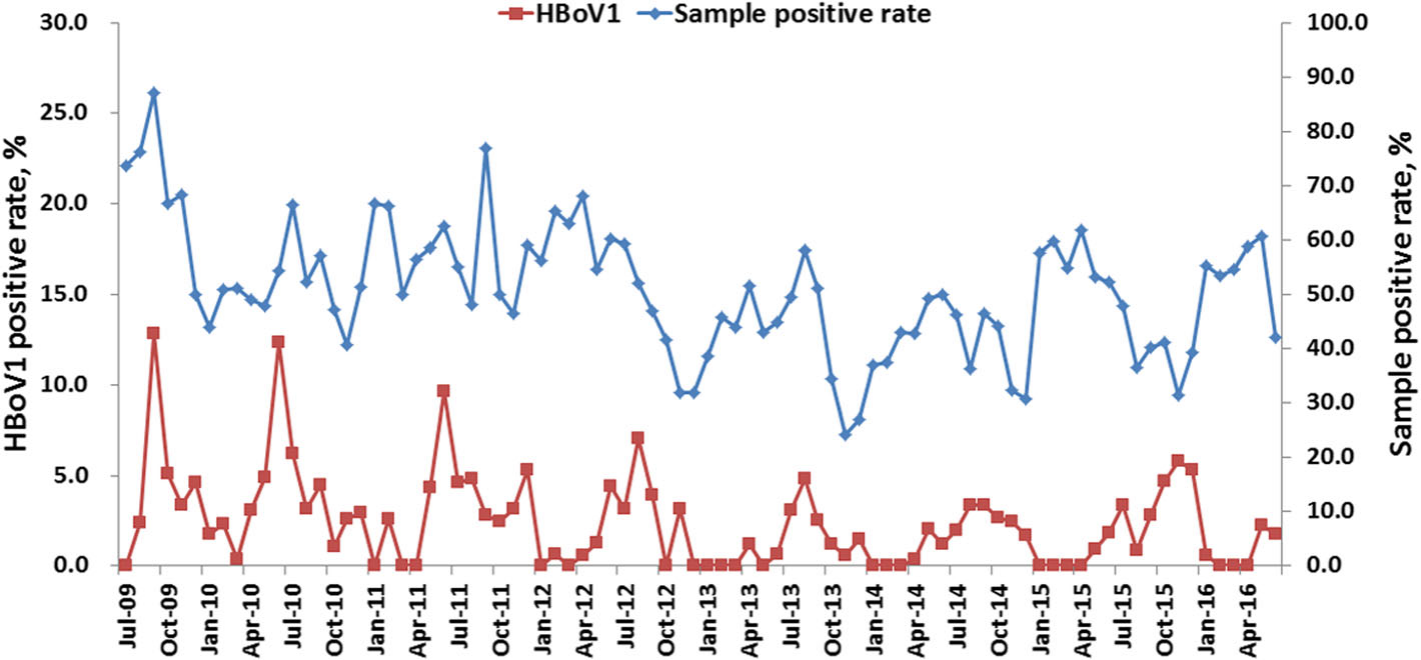
Seasonal distribution of HBoV1 infection in pediatric patients hospitalized with acute respiratory illness from July 2009 to June 2016

In this study, we monitored the prevalence of HBoV1 in patients (≤14 years old) hospitalized with ARI from July 2009 and June 2016. Overall, there were two main prevalence peaks in each year. The large peaks of the epidemic were between June and September of each year, including September 2009 (12.8%, 20/156), June 2010 (12.3%, 10/81), June 2011 (9.6%, 10/104), August 2012 (7.0%, 10/142), August 2013 (4.8%, 10/208) and September 2014 (3.4%, 5/148). The small prevalence peaks were between November and December of each year, including December 2009 (4.6%, 5/108), December 2010 (3.0%, 3/101), December 2011 (5.3%, 7/132), November 2012 (3.1%, 5/160) and December 2013 (1.5%, 3/200). Conversely, in 2015 the large and small prevalence peaks were November 2015 (5.8%, 7/121) and July (3.4%, 4/119), respectively. There was no obvious cut off between the large and small prevalence peaks in 2014 (Fig. 2). The same temporal distribution was observed between the monthly distribution of HBoV1 and proportion of positive samples (Fig. 2).

### Correlation between HBoV1 epidemics and meteorological conditions

We collected meteorological data for Guangzhou, including monthly mean temperature, mean relative humidity, rainfall, mean wind speed, mean air pressure, mean vapor pressure and sunshine duration for a 7-year period, to explore the correlation between meteorological conditions and prevalence of HBoV1.

**Fig. 3:**
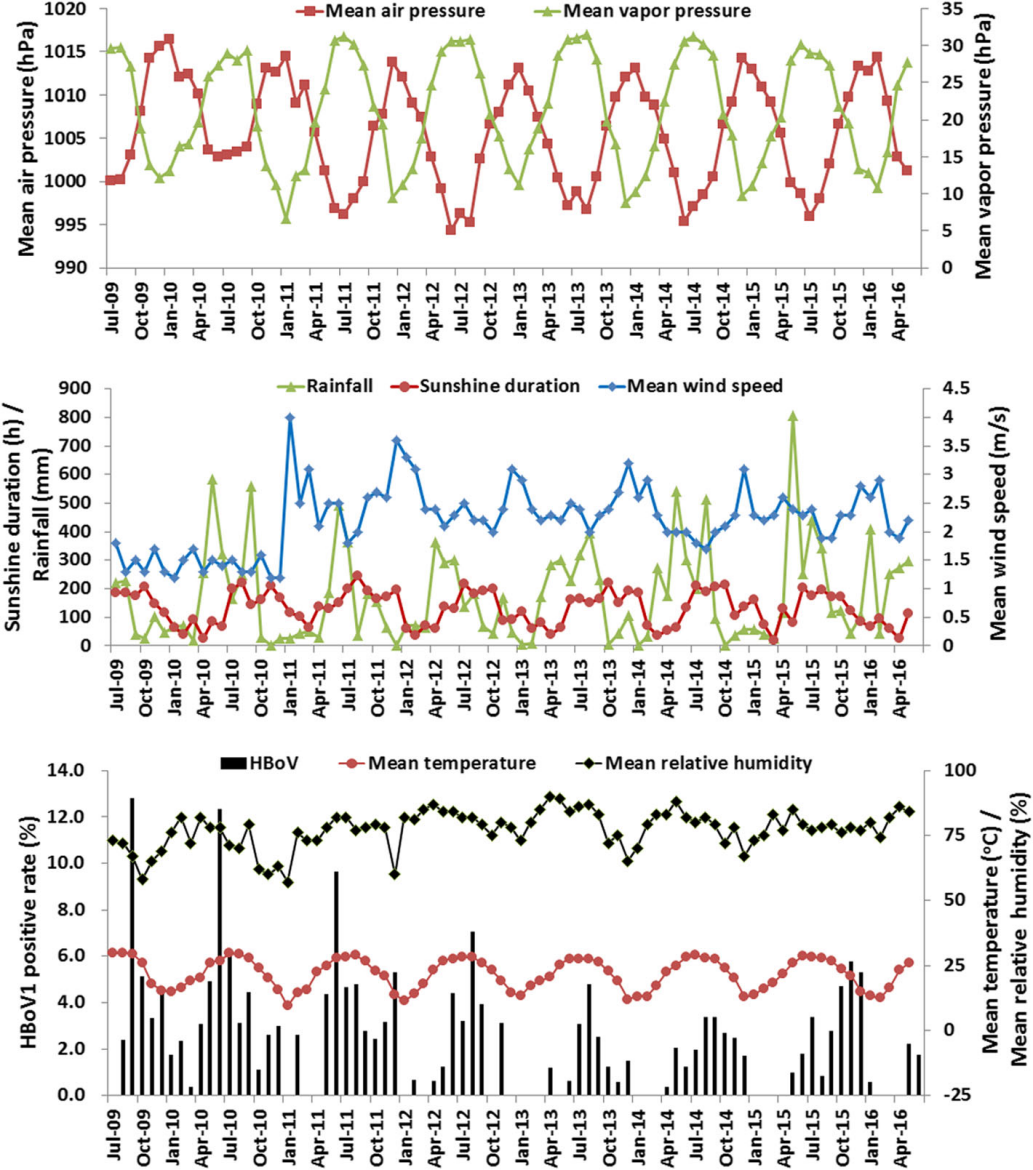
Monthly distribution of HBoV1 and meteorological factors in Guangzhou from July 2009 to June 2016

Guangzhou, which is located in southern China (longitude 112° 57′ to 114° 3′, latitude 22° 26′ to 23° 56′), has a maritime subtropical monsoon climate. Between July 2009 and June 2016, the mean temperature was 21.8 ± 5.8 °C (mean ± standard deviation), humidity was 77.2 ± 7.3%, sunshine duration was 132.7 ± 59.5 h, wind speed was 2.2 ± 0.6 m/s, rainfall was 175.2 ± 165.9 mm, air pressure was 1005.6 ± 6.0 hPa and vapor pressure was 21.3 h ± 7.4 hPa. Between 2009 and 2016, the mean temperature from May to September was greater than 25 °C (Fig. 3).

For multiple linear regression analysis of HBoV1 prevalence and meteorological conditions correlation, independent variables of mean air pressure (adjusted R^2^ = 0.793, *p* < 0.001) and mean vapor pressure (adjusted R^2^ = 0.929, *p* < 0.001), which linearly associated with mean temperature, and rainfall (adjusted R^2^ = 0.278, *p* < 0.001), which strongly correlated with mean relative humidity, were excluded. The independent variables for the final multiple linear regression analysis included mean temperature, mean relative humidity, mean wind speed and sunshine hours. The effect of temperature had a delay therefore mean temperature in the preceding month (mean temperature 1 month before) was also included as an independent variable in the analysis (Table 2). Both regression models were established (*p* < 0.001) and the adjusted R^2^ values were 0.373 and 0.231 in the mean temperature in the preceding month model and the current monthly temperature model, respectively. HBoV1 prevalence was positively correlated with temperature (coefficient = 0.259 in the current temperature model (*p* = 0.002), coefficient = 0.328 in mean temperature in the preceding month model (*p* < 0.001)). Conversely, HBoV1 prevalence was negatively correlated with relative humidity (coefficient = − 0.126 in the current temperature model (*p* = 0.024), coefficient = − 0.083 in the temperature delay model (*p* = 0.039)) (Table 2).

**Table 2:** Multiple linear regression analysis of the correlation between HBoV1 prevalence and meteorological factors in Guangzhou from July 2009 to June 2016

Meteorological factors	Correlation coefficient	*p* value
Mean temperature (°C)	**0.259**	**0.002**
Mean relative humidity (%)	**−0.126**	**0.024**
Mean wind speed (m/s)	−0.190	0.736
Sunshine duration (h)	−0.006	0.426
The regression model adjusted R^2^ = 0.231, *p* < 0.001		
Mean temperature in the preceding month (°C)	**0.328**	**< 0.001**
Mean relative humidity (%)	**−0.083**	**0.039**
Mean wind speed (m/s)	−0.453	0.317
Sunshine duration (h)	−0.014	0.051

Multiple linear regression analysis was performed using HBoV1 monthly prevalence as the dependent variable, monthly mean temperature (or mean temperature in the preceding month), mean relative humidity, mean wind speed and sunshine duration as the independent variables

Data captured in bold are highly significant

## Discussion

ARI is one of the most common human diseases, predominantly caused by different respiratory viruses [41, 42]. One of these viruses, HBoV1 infection, causes global epidemics, has a high public health burden and circulates with different patterns in different areas [3–7, 43]. In general, the prevalence of viruses varies because of factors such as geographical location, climatic conditions, population and social activity [38]. Epidemiology of HBoV1 in temperate regions has been described in more detail and a high incidence of infection has been observed in children under the age of 2 years in winter and spring [15, 16, 39, 44].

To describe the epidemiology of HBoV1 in Guangzhou, we collected throat swabs from 11,399 children (≤14 years old), hospitalized with ARI and monitored HBoV1 and other common respiratory pathogens over a 7-year period (Table 1).

In the current study, 86.5% (9857/11399) of patients were under the age of 5 years, with a median age of 1.75 years, indicating that infants and young children were most at risk of ARI, consistent with previous reports [45, 46]. Overall, 49.2% (5606/11399) of patients tested positive for one or more respiratory pathogens, 2.2% (248/11399) of patients were tested with HBoV1 infection (Table 1). A higher prevalence of HBoV1 was detected in male patients compared with female patients (*p* = 0.019), consistent with previous reports [15, 16, 39, 44].

Co-infection with HBoV1 and other pathogens is common [14, 15]. In our study, 45.2% (112/248) of HBoV1-positive patients also tested positive for other pathogens (Table 1). This may be partly caused by coinciding epidemics of HBoV1 and other pathogens. In our study, the HBoV1 seasonal distribution and total positive pathogen distribution were consistent, confirming this inference (Fig. 2). Current research shows that HBoV1 infection can lead to the collapse of the first line of defense of airway epithelium [35–37], which may lead to a higher susceptibility to other pathogens, explaining the high rate of co-infection. Whether co-infection leads to more severe disease is currently unknown and more research is needed to determine this. The characteristics of the HBoV1 infection are likely to be a good model for studying the effects of co-infections.

In this study, there was a significant difference in prevalence of HBoV1 in patients of different ages (*p* < 0.001). The majority of HBoV1 infections occurred in patients under 2 years old and the peak frequency of HBoV1 infection occurred in patients aged 7–12 months (Fig. 1), consistent with previous serological and epidemiological reports on the virus [8–11, 15, 16, 39, 44]. This might be because children’s immune systems are still under development and maternal antibodies gradually disappear in this age group. The distribution of HBoV1 in patients of different ages will provide important reference for future vaccines and new drug research and development, as well as providing important data for disease prevention and control.

Many factors affect the epidemiology of pathogens, such as geographical location and local climate. Guangzhou, a central city and main transport hub in southern China, is located in a subtropical region. Guangzhou is hot and has high annual rainfall, long summers, short winters and the annual precipitation and high temperature are almost in the same period (Fig. 3). In this study, two HBoV1 peaks were observed (Fig. 2). The large prevalence peaks of HBoV1 infection occurred between June and September of each year, which are the summer months in Guangzhou, with mean temperatures of higher than 25 °C (Fig. 3). Small peaks of HBoV1 infection occurred in winter, between November and December of each year. This seasonal distribution is similar to the prevalence in subtropical regions reported previously [47], but different from the HBoV1 epidemics in temperate regions, which mostly occur in winter and spring [15, 16, 39, 44], as well as from tropical regions, such as India, where no obvious epidemic season has been found [48].

To analyze the correlation between HBoV1 prevalence and meteorological conditions, multiple linear regression analysis was performed, with HBoV1 monthly prevalence as the dependent variable and mean temperature (or mean temperature in the preceding month), mean relative humidity, mean wind speed and sunshine duration as the independent variables (Table 2). Both regression models were established (*p* < 0.001) and the adjusted R^2^ value (0.373) of the temperature dorp 1 month model was greater than the adjusted R^2^ value (0.231) of the current monthly temperature model, indicating that the temperature dorp 1 month model had better explanatory power than the current monthly temperature model. Both of the models showed that the prevalence of HBoV1 was significantly correlated with temperature and relative humidity (Table 2). In detail, HBoV1 prevalence was positively correlated with temperature, that is consistent with previous reports [47, 49]. Conversely, HBoV1 prevalence was negatively correlated with relative humidity, this was different from a previous report in Suzhou [47], which may be related to Guangzhou high humidity (mean monthly relative humidity was 77.2 ± 7.3%) (Fig. 3). It is common for pathogen prevalence to fluctuate over time because of a variety factors. In this study, HBoV1 prevalence was relatively low in 2013 to 2014. It might be partly related to the relatively higher mean relative humidity during this period (Fig. 3). Climate conditions may impact the survival and spread of respiratory viruses, however no significant linear relationship between HBoV1 infection and wind speed or sunshine duration were found in this study (*p* > 0.05) (Table 2).

Some limitations of this study should be noted. First, because our study mainly focused on HBoV1 circulation in hospitalized patients with ARI, HBoV1 in outpatients and the asymptomatic population were not included. Second, many factors can affect virus epidemics, meteorological data analysis alone may not serve as a final conclusive interpretation. Third, the study was only conducted in three hospitals and may not be representative of the overall population.

## Conclusions

Our study has provided a better understanding of the epidemiology of HBoV1 in subtropical regions, specifically correlations with climate data; these data will be helpful for future control and prevention of HBoV1 infections.
